# Citizens’ Perception and Concerns on Chemical Exposures and Human Biomonitoring—Results from a Harmonized Qualitative Study in Seven European Countries

**DOI:** 10.3390/ijerph19116414

**Published:** 2022-05-25

**Authors:** Linda Matisāne, Lisbeth E. Knudsen, Joana Lobo Vicente, Maria Uhl, Andromachi Katsonouri, Annick D. van den Brand, Tamar Berman, Mirjana Dimovska, Eleni Anastasi, Anthi Thoma, Szilvia Középesy, Dragan Gjorgjev, Mirjana Borota Popovska, Shalenie P. den Braver-Sewradj, Tamás Szigeti, Marija Topuzovska Latkovikj, Inese Mārtiņsone, Lāsma Akūlova, Linda Paegle

**Affiliations:** 1 Institute of Occupational Safety and Environmental Health, Rīga Stradiņš University, Dzirciema 16, LV-1007 Riga, Latvia; inese.martinsone@rsu.lv (I.M.); lasma.akulova@rsu.lv (L.A.); linda.paegle@rsu.lv (L.P.); 2 Department of Public Health, University of Copenhagen, Øster Farimagsgade 5, 1014 Copenhagen, Denmark; liek@sund.ku.dk; 3 European Environment Agency, Kongens Nytorv 6, 1050 Copenhagen, Denmark; joana.lobo@eea.europa.eu; 4 Environment Agency Austria, 1090 Vienna, Austria; maria.uhl@umweltbundesamt.at; 5 State General Laboratory, Ministry of Health, P.O. Box 28648, Nicosia 2081, Cyprus; akatsonouri@sgl.moh.gov.cy (A.K.); eanastasi@sgl.moh.gov.cy (E.A.); cthoma@sgl.moh.gov.cy (A.T.); 6 National Institute of Public Health and the Environment (RIVM), P.O. Box 1, 3720 BA Bilthoven, The Netherlands; annick.van.den.brand@rivm.nl (A.D.v.d.B.); shalenie.den.braver@rivm.nl (S.P.d.B.-S.); 7 Department of Environmental Health, Ministry of Health, King David Street 20, Jerusalem 91010, Israel; tamar.berman@moh.gov.il; 8 Institute of Public Health of the Republic of North Macedonia, 1000 Skopje, North Macedonia; mirjana.dimovska@medf.ukim.edu.mk (M.D.); dgjorgjev@gmail.com (D.G.); 9 National Public Health Center, Pf. 839, 1437 Budapest, Hungary; kozepesy.szilvia@nnk.gov.hu (S.K.); szigeti.tamas@nnk.gov.hu (T.S.); 10 Institute for Sociological, Political and Juridical Research, 1000 Skopje, North Macedonia; mborota@isppi.ukim.edu.mk (M.B.P.); marija_t@isppi.ukim.edu.mk (M.T.L.)

**Keywords:** human biomonitoring, focus group, citizen reflections, chemical exposure, HBM4EU

## Abstract

Exposure to different chemicals is an inevitable part of our everyday lives. Within HBM4EU, focus group discussions were conducted to gather data on citizens’ perceptions of chemical exposure and human biomonitoring. These discussions were hosted in Cyprus, Denmark, Hungary, Israel, Latvia, the Netherlands, and North Macedonia following a protocol developed in the first round of discussions. Results indicate the very high concern of European citizens regarding food safety and the environment. Focus group participants were well aware of potential uptake of chemicals through food consumption (e.g., preservatives, flavor enhancers, coloring agents, pesticides, fertilizers, metals), drinking water, or from polluted air and water. One of the positive aspects identified here, is the high interest of citizens in awareness and education on personal measures to control exposure. The promotion of personal behavioral changes requires active involvement of society (e.g., commuting habits, energy choices, waste disposal, dietary habits). Activities should focus on raising awareness of the general public, implementation of policy measures, and mainstreaming of related topics into the education system. Raising awareness of the general public may promote engagement of citizens, which in turn may empower them to put pressure on politicians to take effective actions. There is also a need for further research which might focus on the impact of country-specific situations and of the COVID-19 pandemic on the exposure of citizens to chemicals.

## 1. Introduction

Exposure to different chemicals is an inevitable part of our everyday lives. On the one hand, the use of chemicals like cosmetics and cleaning agents makes life easier and more convenient or pleasant, but on the other hand, the individuals using those products are more exposed to chemicals [1]. Unintentional exposure to chemicals is also possible. For example, food may contain chemicals added for a technological purpose (e.g., food additives), residues of pesticides or veterinary drugs, or contaminants arising from environmental pollution of the air, water, and soil [2].

Human biomonitoring is an effective way to quantify people’s exposure to chemicals. It measures the levels of substances in human body fluids and tissues (e.g., blood, urine, saliva, breast milk, hair, nails, adipose tissues, or teeth) arising from exposures to chemicals and contributes to a better understanding of resulting possible adverse health effects and exploration of appropriate preventive actions [3,4,5]. Human biomonitoring reflects the absorption of chemicals from all sources (e.g., water, air, soil, dust, personal care products, and food) and via all uptake routes (inhalation, ingestion, and dermal absorption).

HBM4EU is a five-year European program that uses human biomonitoring to assess human exposure to chemicals in Europe and to inform policymakers for improved protection of European citizens’ health from chemical exposure [6]. To address public concern, citizens’ involvement was considered important to capture not just scientific but also societal and policy considerations in assessing the extent of exposure and the risk of health effects related to chemical exposure. There were two rounds of focus group discussions that were organized to obtain a better understanding of the public’s concern about chemicals and their understanding of human biomonitoring across Europe:

- In Austria, Portugal, Ireland, and the UK in 2018 and 2019 (the results covering analyses of discussions have been published earlier [7] (further in the text referred to as the first round of the HBM4EU focus groups);
- In Cyprus, Denmark, the Netherlands, Hungary, North Macedonia, Israel, and Latvia in 2020 and 2021 (further in the text referred to as the second round of the HBM4EU focus groups).

The objective of the second round of the focus groups of HBM4EU was to improve the geographic representation of citizen feedback in the advancement of European human biomonitoring and to gather additional data to better understand: (1) citizens’ perception of chemical exposure in their daily lives and human biomonitoring; (2) citizens’ concerns regarding exposure to chemicals; (3) beliefs towards chemical exposure and safety, as well as regarding human biomonitoring; (4) the influence of the COVID-19 pandemic on the perception of citizens about the exposure to chemicals.

The last topic was added to the focus group discussions as the second round of the discussions was performed during the COVID-19 pandemic and we thought it is essential to find out if there are any changes in the citizens’ perception as the COVID-19 pandemic has left an impact on the chemical exposure of the population. However, the impact differed in different stages of the pandemic. As a response to the pandemic, when governments enforced a variety of restrictions on everyday activities (e.g., lockdown), a drop in air pollution was observed [8]. This was caused by less use of vehicles, a decrease in heating due to the closure of workplaces, non-functioning of industries, etc. [9,10]. 

The increased use of disinfectants and sanitizers as part of cleaning practice and hand hygiene has been essential to mitigate the spreading of the SARS-CoV-2 virus. Despite the potential benefits of these chemicals in the fight against this virus, their continuous use and possible misuse, and overuse may lead to short-term and long-term adverse effects on humans and the environment [11]. The impact from people’s exposure to chemicals from disinfectants and sanitizers through skin absorption, inhalation, or hand-to-mouth behavior are under scrutiny [12,13]. Environmental impacts on physical spaces (land, water) from increased waste have also been reported, especially from medical waste such as contaminated masks, gloves, used or expired medications, and other items mixed with domestic waste [9]. 

## 2. Materials and Methods

In 2018 and 2019, a cross-sectional observational qualitative study to collect data through semi-structured focus groups was conducted in four European countries: Austria (February 2018), Portugal (May 2019), Ireland (September 2019), and the UK (October 2019) [7]. To obtain data from other European countries and improve the representation of the different regions of Europe which might have different perceptions of chemical safety, more focus group discussions were organized in 2020 and 2021. These followed the same research principles and focus group guidelines as before. All countries represented in the HBM4EU project were invited to show their interest in hosting focus group discussions. Based on this interest, to achieve a good mix of the countries in terms of size, geographical location and historical development, the Management Board of the HBM4EU project approved organization of the focus group discussions in the following seven countries: Cyprus (October 2020), Denmark (two groups in November 2020), the Netherlands (four groups in November and December 2020), Hungary (December 2020), North Macedonia (June 2021), Israel (July 2021), and Latvia (August 2021).

### 2.1. Participants: Sampling, Inclusion/Exclusion Criteria, Description of Sample

Participants were recruited following a purposive sampling (non-random, conceptually driven) method in each of the seven involved countries. Eligible participants were adult citizens (18+ years old) of both genders. As the focus group discussions were held in the national languages of the participating countries, the persons who could not master the language that the focus group was conducted in (e.g., Hebrew in Israel or Latvian in Latvia, etc.) were excluded from participation in the discussion. Also, participants who were not able to provide written or electronic consent to participate in the focus group were excluded from participation. 

The countries organizing focus group discussions used different methods to recruit a sufficient number of participants. Two of the HBM4EU project partners (in Denmark and the Netherlands) outsourced the recruitment to specialized external service providers. Social media (Facebook and Twitter) posts and groups (WhatsApp) were used in Latvia, Northern Macedonia, and Israel. A personal contact network was used in Hungary and Israel. In Cyprus, focus group participants were recruited by calling randomly formed telephone numbers. In addition, in North Macedonia, several persons were invited based upon their prior knowledge about the topic, and in Latvia an article was published on the national occupational health and safety portal www.stradavesels.lv (accessed 10 March 2022). Most of the countries used various methods to ensure heterogeneity of the focus groups regarding age, educational level, urban/rural residence, and professional background of the participants (e.g., by boosting social media posts or selecting participants from existing databases and networks). Some of the countries (e.g., North Macedonia) also paid attention to other recruitment criteria like prior knowledge of the topic of the focus group discussions, belonging to a category vulnerable to pollutants (people with chronic diseases, pregnant women), or minor ethnic communities in the country. 

No monetary or other compensation was offered for the participation and contribution in several countries; however, monetary incentives were provided in Cyprus (60 Euros per participant), Israel (a voucher—the equivalent of approximately 13 Euros), the Netherlands (35 Euros—provided by the recruitment service) and Denmark (a gift card—the equivalent of approximately 80 Euros). Any expenses related to travel to onsite events were also reimbursed (e.g., in Denmark).

### 2.2. Procedures and Instruments of Data Collection

A standardized procedure was used as the basis for all eleven focus group discussions. Due to the different epidemiological restrictions to mitigate the spreading of the SARS-CoV-2 virus, varying discussion methods were used—two focus groups took place within the facilities of the research units or local institutions and nine focus groups were organized using online platforms—Zoom, GoToMeeting and Brainstork.

Depending on the country and size of the focus group, one or two experienced moderators led the sessions according to written guidelines which consisted of logically proceeding questions. Moderators were supported by a note-taker and a technical support person in case of online discussions. The sessions started with the moderator seeking consent to audio record the discussion, followed by an overview of the main sections to be discussed, main goals and rules for group discussion, a presentation for the participants (warm-up), and a short and brief introduction to the HBM4U project. Afterward, moderators using a non-directive semi-structured approach following the slightly modified guidelines which were used during the first round of the discussions [7]. It included the following topics:

1. Chemicals to which we are exposed in our daily lives (through food, pollution, etc.) -which substances concern us most, to which substances are we more exposed, what are the health problems that can be related to chemicals;
2. The present situation of human biomonitoring—have you ever heard about human biomonitoring, what is done in this area of human biomonitoring, who works in this area, for what and how is it done;
3. Human biomonitoring—how will the results of human biomonitoring and, particularly, of HBM4EU be relevant for the participants, in which areas of personal life can these results be more relevant;
4. The near future of human biomonitoring—which results can be achieved by the time the HBM4EU initiative will have ended, how can these results be used, how would the participants like to be informed about the results, in which way can the results be communicated to the general public, which political measures should be taken, how can the public awareness on human biomonitoring be increased.

The modifications in focus group guidelines included changing the year to be mentioned when talking about the future of the Human Biomonitoring Project HBM4EU, the expected results of this future, and other expectations of the focus group participants regarding biomonitoring. The questions were flexible in terms of order and depth of discussion, so national research teams could reorganize the discussion to reflect local needs. In addition to the topics discussed in 2018 and 2019, several questions related to the impact of the COVID-19 pandemic on chemical exposure in our lives were added for discussions in 2021. They covered aspects related to changes in our habits and every-practices due to the COVID-19 pandemic and their influence on exposure to chemicals.

The duration of the focus groups was variable and ranged between 70 to 180 min each (full session). During the sessions of onsite discussions, participants were offered light refreshments, which also contributed to “breaking the ice” and promoting group interaction. Sometimes, a PowerPoint presentation was shared on the screen when the relevant questions were discussed among online participants. Participants of online discussions (e.g., in Latvia, Israel) also wrote comments in the chat during the sessions, which were later incorporated into the transcripts.

All the focus group sessions were recorded with permission from the participants. These recordings were later used for full transcripts and to match the information correctly. Recordings were uploaded and kept on the internal protected servers of HMB4EU project partners, taking into account the General Data Protection Rules (GDPR), and will be deleted according to the national requirements.

### 2.3. Data Analysis

Anonymized transcripts were prepared in the language which was used during the discussions, including careful de-identification of all participants. Content analysis of transcripts was provided by two independent and experienced researchers of each country, following the grounded theory principle [14,15]. Each of the researchers independently read through the transcripts, noting keywords or phrases (open coding) which were used for constructing a coding framework of similar categories. The coding framework was triangulated for interpretative validity by the moderators and co-moderators of the focus groups [16]. The developed framework was then used to identify key themes arising from the data gathered during all of the discussions. In the final stage, an experienced researcher in each country reviewed the relevant transcripts to verify the findings and translate them into English. The results of the coding by country are presented in Table A1 included in Appendix A. Only the codes mentioned in more than one country are included in this table.

The results of all countries were summarized and the most relevant quotes from the focus group sessions were marked at all stages of the analysis of transcripts. After that, the researchers (L.M., L.K.) selected and suggested to other authors the examples-quotes that best explain the different answers to the questions and demonstrate various opinions and experiences. The final set of quotes was created by a simple agreement between all authors. In the section of the results, only one or two supporting quotes are provided, other relevant quotes are given in Appendix A (Table A1).

### 2.4. Ethical Issues

Before starting the discussions within the focus groups, all of the participants signed an informed consent form in a written or electronic manner. Within the consent form, it was explained that the participation was voluntary, that the participants were free to withdraw their participation at any time and that all of the collected data were anonymous and confidential. In addition, the moderator of each focus group explained to the participants the purpose of the study and allowed the participants to ask questions.

The process and guidelines of the focus group discussions within the HBM4EU project were approved by the project management board. Adherence to the rules of the GDPR was assured by the national project partners carrying out the discussions by national legal acts. In all focus groups, participants gave informed consent through a declaration of consent on data protection of the affected persons (according to Art 6 para 1 lit a GDPR) Citizen Workshop on Human-Biomonitoring in the EU. In Denmark, the study protocol in Danish accompanied by the informed consent form and invitation letter was approved by the institutional ethics committee at the Faculty of Health and Medical Sciences (504-0208/20-5000) and by the institutional data processor (514-0544/20-3000) at University of Copenhagen. A controller-to-controller data transfer agreement was signed between Norstat and the University of Copenhagen. Additionally, in Latvia, positive approval for the conduction of the focus group was received from the Ethics Committee of Rīga Stradiņš University (protocol No. 22-2/250/2021, 14 April 2021). According to the national legal requirements, approval from the ethics committee was not required in the other countries organizing focus group discussions in 2020 and 2021.

## 3. Results

A total of eleven focus groups in seven countries were undertaken (for details see Table 1). The number of participants varied between three (in The Netherlands) and twelve (in North Macedonia) per session.

In total, 78 persons took part in the focus group discussions (41 females (53%) and 37 males (47%), aged between 18 and 74). Sample characterization, in terms of sociodemographic variables, by country, is provided in Table 2.

Five main themes (categories) of the second round of focus group discussions were revealed: (1) perception of chemical risks and their impact on human health; (2) present situation of human biomonitoring; (3) expectations from human biomonitoring and particularly HBM4EU; (4) communication of the results of human biomonitoring and HBM4EU; and (5) perception of the influence of the COVID-19 pandemic on exposure to chemicals (see Table 3).

### 3.1. Perception of Chemical Risks and Their Impact on Human Health

When addressing the perception of focus group participants on chemical risks and their impact on human health, three sub-categories were identified: (a) main concerns regarding exposure and health effects; (b) concerns regarding harmful substances with the most exposure; and (c) aspects related to personal behavior.

#### 3.1.1. Main Concerns Regarding Exposure and Health Effects

Focus group participants were asked if they had concerns about exposure to a variety of chemicals and associated health risks. The biggest concern was expressed about exposure to chemicals from the consumption of food (e.g., preservatives, flavor enhancers, coloring agents). Air pollution and chemicals in drinking water were also mentioned by focus group participants, however, less often. Some of the participants were not able to name a specific problem (just general environmental pollution was mentioned), while others wanted to highlight air pollution from traffic (e.g., in Denmark pollution from traffic during biking was mentioned), heavy industry, and combustion processes.

*“Food is another source of exposure to chemicals and as an example, I would like to mention the consumption of the food color that is found in E101 (sunset yellow) juices …. This is a very carcinogenic chemical”*

(North Macedonia)

The location of industrial facilities close to the living area or living in a polluted area was mentioned in the focus group from the Southern countries (Cyprus, Israel, North Macedonia). The same countries, except Israel, also stressed contamination of soil arising from the use of pesticides or herbicides, which might be related to the economic activities typical for Southern Europe. Focus group participants of both non-EU member countries (Israel and North Macedonia) also discussed the storage of hazardous chemicals including illegal waste disposal, illegal waste burning, chemicals and waste left in closed factories.

*“As a resident of Haifa Bay [polluted are in Israel], I am very aware of air pollution problems and think human biomonitoring is relevant to my life”*

(Israel)

In the Netherlands, chemicals in personal care products were also considered of concern. In other countries, there was less concern about this source of exposure. In some cases, chemicals and dust in the workplace, specific work with building materials, exposure from several sources, chemicals from plastic toys, and use of packaging were also mentioned, however, this concern can be characterized as minor.

When asking the participants which exposure routes concern them the most, the focus group participants were able to specify all main exposure pathways: oral uptake, inhalation of contaminated air, and exposure through skin contact were mentioned by some focus group participants. In addition, in Denmark, several of the participants were not able to highlight which substances they are more exposed to. They stressed that exposure to substances comes from several sources and that it is difficult to choose which one is the main exposure.

*“I simply believe that it is all of it. I think it can be our clothes, the way it is produced, the substances in it, what we wash it in, the food we consume, it can be something in the air and ordinary pollution from factories”*

(Denmark)

Another concern in the discussion was the consequence of being exposed to chemicals. In total, seventeen different health effects arising from exposure to chemicals were mentioned during the focus group discussions. In general, long-term health effects were of the biggest concern. Cancer was highlighted in more than half of the focus groups. Allergies, including asthma, skin problems, respiratory diseases, and the effect of chemicals on fertility were also reported several times. Hypersensitivity, chronic obstructive pulmonary disease, diabetes, poisoning, eye irritation, and nervous disease—all of them were only mentioned by one focus group participant.

*“On account of serious health issues such as cancer… especially with regards to what we eat and how we eat it.”*

(Cyprus)

The issues, which dominated the participants’ interest could be classified as “health effects due to chemical exposures” and to “personal risk management actions”. ‘Well-known’ diseases (e.g., cancer), especially previous personal or family experience, and unknown consequences of being exposed to chemicals can be given as examples of health-related factors. Availability of healthier alternatives/choices (e.g., to choose healthier food, to choose a holiday destination with lower air pollution) and the possibility to influence exposure (“*you can more influence what you eat if compared to what you breathe*”) are factors to be highlighted as examples for personal behavior. In addition, exposure to chemicals at the workplace was also mentioned by one focus group participant.

*“I mean, when you go somewhere with your whole family every year, you would want to know, is the air, is the food, the environment there healthy? If you go there on vacation every year, you can also choose for another destination”*

(The Netherlands)

During the discussions in Latvia and Cyprus, the increased vulnerability of specific population segments was brought up. The following population segments were mentioned: pregnant women, children, persons with chronic diseases or underlying illnesses, persons with unhealthy eating habits, persons employed in certain professions (e.g., in factories or health care), elderly persons, persons with special needs, and persons living in polluted areas.

#### 3.1.2. Concerns Regarding Harmful Substances with the Most Exposure

A large number of different chemicals were mentioned while discussing harmful substances with the most exposure. Food preservatives, flavor enhancers, coloring agents, and food additives (‘E numbers’) were the main concern of most focus group participants in four different countries (Denmark, The Netherlands, North Macedonia, and Latvia). 

The second most-often mentioned concern was related to pesticides, herbicides, and insecticides which were highlighted in five countries. However, when compared with chemicals in food, this group is of less concern. Also, the use of fertilizers was mentioned by two countries. In general, these results also stress the high concern of Europeans regarding contaminated food chains, as fertilizers and pesticides are used in agricultural production.

*“Pesticides can contaminate our food, the water and the environment... I remember reading as a student in Mytilene [Greece] about a study, which showed that the pesticides sprayed on the local olive groves contaminated the soil and water bodies and that the contamination persisted for a long time. I mean, the cycle of pesticides ‘use and effects’ is unbelievable”*

(Cyprus)

The next most frequently discussed group of chemicals was heavy metals, but the specific metals that were mentioned differed between countries (in Cyprus-mercury, in Hungary-lead, in North Macedonia-hexavalent chromium). However, also, in this case, oral uptake through food or food supplements was the exposure route of main concern.

*“I am very concerned about heavy metals in food supplements. I don’t think this is sufficiently regulated”*

(Israel)

Dioxins and polychlorinated biphenyls were discussed in two countries (North Macedonia, and Latvia). The following groups of products were mentioned by the two countries: cleaning products, detergents, cosmetics, pharmaceutical drugs, medication, smoking and tobacco products, substances released during the combustion process, and petroleum products. In addition, the following chemicals or chemical products were noted only in one country: shampoos, plastics, garments, and shoes containing harmful substances, hormone-disrupting substances, parabens, PM (PM_10_ and PM_2.5_) particles, methanol, benzene, toluene, xylene, acrylonitrile, methyl acrylate, formaldehyde, asbestos.

#### 3.1.3. Aspects Related to Personal Behavior

Individual efforts to avoid chemicals were discussed in focus groups from three countries: Cyprus, Denmark, and the Netherlands. Only consuming biological and organic food was mentioned in all these countries, however, the context differed. For example, focus group participants in Cyprus acknowledged that they grow such food themselves, but participants in Denmark indicated purchasing biological and organic food. Participants in Denmark also stressed that one of the factors that can influence their choice is the price level of ecology products.

*“When I see that I can get a food that costs roughly the same and which is organic, then I buy the organic if there is not that big a price difference”*

(Denmark)

Several other food-related aspects were given as examples for explaining personal behavior to reduce exposure to chemicals: getting fresh food, active avoidance of food additives (E-numbers), cleaning fruit before consumption, and use of sweeteners to replace sugar. Some other participants mentioned opting for hand-made cosmetics/ soaps and buying particular types of personal care products.

### 3.2. Present Situation of Human Biomonitoring 

When addressing perception and understanding of human biomonitoring and the HBM4EU initiative, the results can be classified into two categories: (a) familiarity with human biomonitoring and HBM4EU; and (b) actors in human biomonitoring.

#### 3.2.1. Being Familiar with Human Biomonitoring and HBM4EU

The general understanding of human biomonitoring in almost all focus groups can be characterized as very low and limited, some of the participants clearly stated that they had never heard of human biomonitoring before and had not done any research before the focus group, and were curious about the meaning of it. Others were giving examples of monitoring of different health parameters (e.g., blood pressure), however, in fact, these answers did not cover the measurement of chemicals in the samples taken from the human body. One of the participants mentioned that he is aware of the term “human biomonitoring” because of the field of his/her studies in the university.

*“That what comes into my mind on human biomonitoring at once is … more related with medical parameters, for example, the oxygen concentration in blood, or blood pressure, or something like that”*

(Latvia)

After a short presentation of the topic, the participants could recall information that human biomonitoring is related to the analysis of biological samples from the human body. Focus group participants in Cyprus, Denmark, and North Macedonia could relate the topic with analysis of blood, in Cyprus and North Macedonia, with analysis of urine. Analysis of semen and hair were mentioned in Denmark. When looking at the type of analysis, monitoring of the presence and/or limits of chemicals was mentioned in three countries (Denmark, North Macedonia, and Cyprus). A total of two countries also mentioned the so-called cocktail effect when effects of exposure to several chemicals at the same time is researched (Denmark and the Netherlands).

*“… for example, examinations of heavy metals in samples of human origin”*

(North Macedonia).

#### 3.2.2. Actors in Human Biomonitoring

In all countries, where the focus group discussions covered topics related to actors who play a role in human biomonitoring, participants stated that multiple professionals with various backgrounds should be involved. Among the key-actors, state authorities in the field of health were mentioned (participants in Cyprus also mentioned institutions working in the field of education). National research communities with scientific institutions and researchers from different scientific fields were the focus of discussions. Chemists, biochemists, biologists, experts in biotechnology, toxicologists, physicians, social scientists, nature scientists, psychologists, and sociologists were given as examples to be involved in the processes related to human biomonitoring.

*“It shouldn’t be only one type of competent experts, who are responsible, but a combination of different experts, such as sociologists, biochemists, and health professionals…”*

(Cyprus)

In several cases, practitioners were also mentioned, including public health workers, nurses, doctors, other workers in hospitals, occupational hygienists, etc. In the case of human biomonitoring and employers, a few focus group participants also stressed their concerns related to data protection. Although some of the focus group participants mentioned the involvement of representatives of specific industries (pharmacy industry or water service industry), other participants stated that the data should be extremely carefully protected, for companies with commercial interests, or even criminals. There were also focus group participants that advised not to involve private organizations in human biomonitoring studies, due to possible data privacy issues.

*“.., you really should prevent misuse [of HBM data], for example by insurance companies or others who could possibly (mis)use these data for their own purposes.”*

(The Netherlands)

In addition to the national level key actors, international organizations were also discussed in Denmark—the World Health Organization, the European Union, and its institutions (e.g., the European Environment Agency). Politicians were mentioned in some focus groups, and the results on policy-related actions are described below.

### 3.3. Expectations from Human Biomonitoring and Particularly HBM4EU

When participants were invited to provide specific expectations from the human biomonitoring process and suggestions about the expected output from the HBM4EU project, a vivid discussion took place, and their suggestions covered a wide range. In five countries policy-related actions were mentioned most often. They include the role of policy decisions driven both at the European and national levels. Getting more control and legislation in the field were also discussed in several countries, however, the term “field” was used in different contexts. For example, in Cyprus and Denmark the focus of the discussions was the prohibition of chemicals/products, in Hungary—food control, but in Latvia—food and construction materials as well as the school environment.

*“Yes, I like such studies…. [they should] serve as a base to improve legislation and the guidelines or some other things in many areas… in the area of medicine and food… An important aspect is the education of the society, I think, that would be one, if not the result, then at least the aim of this project”*

(Latvia)

The focus group participants expect also better research in the field and human biomonitoring done systematically (e.g., 2–5 year cycles—mentioned in Cyprus), improved laboratory capacity, and scientific debate (discussed in North Macedonia). In addition, depending on the country, cooperation with the involved parties (scientists, other professionals, and politicians) should be established or improved.

*“This is a good step for cooperation between science and politics. This project ‘diagnoses’ the condition, which is the most important, and it leads to successful ‘treatment’”*

(North Macedonia)

When looking at specific areas, labeling of products containing chemicals was discussed most, and the discussions covered topics related to both—better labeling and more information on labeling. Consumer products (highlighted in Cyprus and Hungary) and foodstuff (highlighted in Hungary) were groups of products mentioned most. Some other focus group participants expect expansion of the list and range of hazardous chemicals to be measured/monitored (mentioned in North Macedonia and Latvia). Actions to be taken at the European Union level covered the need for standardized limit values with the highest common denominator for the entire European Union, increased European standards, and improved standardized EU labeling schemes.

*“The case is that if a product is approved in EU- countries, then it can be sold in all countries. So, of course, it is important to raise the standards for the entire EU”)*

(Denmark)

Among expressed expectations, some other interesting initiatives were suggested. For example, mainstreaming of the relevant topics in school curricula was mentioned in Cyprus and the Netherlands, implementation of taxation—in Cyprus, a designated area in a supermarket—in Denmark.

*“Human biomonitoring should be introduced to high-school students as part of a lesson or seminar. For example, we were introduced to the importance of blood donations as secondary-school students. Since then, we became blood donors, again voluntarily. So, the message is delivered to the student at that age, then it will be easier for him/her to participate in human biomonitoring studies in the future”*

(Cyprus)

Finally, improved protection of vulnerable groups (including a better understanding of their exposures) is expected in Cyprus and the Netherlands. In the Netherlands, it was also discussed whether the participants would participate in a human biomonitoring study, and why. The participants indicated that intrinsic interest in the topic and the societal relevance of such studies are the main drivers to participate. They would also prefer feedback on their personal results. However, there are limits to the efforts people are willing to make. For example, a few participants indicated that providing blood samples on a daily basis was considered a bridge too far.

### 3.4. Communication of the Results of Human Biomonitoring and HBM4EU

When addressing communication on human biomonitoring and the HBM4EU initiative, the results can be classified into two categories: (a) content of the communication and (b) communication channels and methods to be used for raising awareness of the general public.

#### 3.4.1. Content of Communication

Similarly, to the focus groups carried out in 2018 and 2019, the results of the focus groups of 2020 and 2021 stress the need for raising awareness of the general public on chemical exposure, human biomonitoring, and HBM4EU, as it may promote citizen engagement, which in turn may empower them to put pressure on politicians to take effective actions.

*“…it is very important to raise awareness among the public and maybe then they could put pressure on policymakers. It is very difficult to achieve anything without political support”*

(Hungary)

Most of the focus group participants agreed that the communication should be understandable, with great transparency and clarity, avoiding scientific jargon, and provided in a non-scientific manner.

*“I also think it would be best if it was published in e.g., an article in the media or something with source references to this report. So, kind of … understandable for the common man who is not a researcher”*

(Denmark)

However, when looking in detail at the main messages, the ideas of the focus group participants varied. Some countries mentioned dissemination of selected and targeted information, e.g., in Cyprus-sector-specific information, in Hungary—only information on most harmful chemicals, but in North Macedonia—information to parents on issues related to children’s health. Personalized messages and the context of influencing personal behavior (personal choice) were discussed in Cyprus, Israel, and the Netherlands.

*“It is important to target young children and increase awareness about environmental health. As an environmental activist, we targeted schools and kindergartens in messaging about single-use plastic and have been successful”*

(Israel)

In addition, it should be pointed out that the suggested communication approach for dissemination of the results was very diverse. Participants in some countries (Hungary and Israel) advised the use of positive messages and avoidance of scaremongering. But in other countries, it was recommended the use of intense, powerful communication that almost shocks people (Cyprus, Hungary) should be considered.

*“…. I’d disseminate positive information as clearly and strikingly as possible. In the long-term, a label should be used that would get a (coherent) E-label and inform us if we bought the healthiest or the least harmful product… Definitely, a positive campaign is needed”*

(Hungary)

When discussing the HBM4EU project, the availability of detailed procedural information about the HBM4EU project was mentioned in Cyprus and the Netherlands. This was considered specifically important when participants are involved in a human biomonitoring study themselves. In the Netherlands, it was also considered very important that the results of HBM studies are publicly available.

#### 3.4.2. Communication Channels and Methods

The depth of the discussion on communication channels and methods of information sharing of human biomonitoring varied across focus groups, with some participants referring to generic types of communication (such as websites, mass media campaigns, social media) whereas others mentioned specific types of actions and tools. The mass media campaign, including the use of regular media as well as social media (Facebook, Twitter, Instagram, YouTube, and even TikTok) were mentioned in most of the countries, and participants from two countries (Cyprus and Hungary) stressed the need of simultaneous use of different channels with visual and graphical display of results (infographics, videos). Some of the focus group participants also discussed key actors in the communications, however, no common tendencies were observed. Among the groups to be involved in spreading messages on chemical exposures and human biomonitoring, non-governmental organizations, research institutions, and even celebrities were mentioned.

*“A component of the follow-up project should provide communication, information, and dissemination of results, in the form of a media campaign, with involvement of the NGO sector”*

(North Macedonia)

Another aspect discussed by the focus group participants was expectations about the communication from governments. There were two opposite opinions; Danish and North Macedonian representatives expect that communication from the governments should be more and of higher quality, but Latvian representatives expressed that the governments should not be involved.

*“… information should not come from the top, from the government, … it should more come from “side”…”*

(Latvia)

In addition to the traditional campaigns, two types of non-traditional activities were identified. Development of special apps, tools, or kits was mentioned in more than half of the discussions, but the examples of the content differed. Representatives of the Cypriot focus group mentioned an app to derive a personalized risk profile, Danish focus groups mentioned scanning consumer products, but in Hungary and Israel labelling of cosmetics and other consumer products to include information on toxic chemicals was suggested. Organizing special events was mentioned in three countries, but each of the events was different. Thus, in Cyprus it was an annual festival or event lasting 2 to 3 days, in Hungary, health days organized in schools, and in Israel, a competition where children, students, and adults could make suggestions for reducing exposure to toxic chemicals.

### 3.5. Perception of Influence of the COVID-19 Pandemic on Exposure to Chemicals

The opinion of focus group participants about the influence of the COVID-19 pandemic on exposure to chemicals was available from three countries: Hungary, Israel, and Latvia. In all countries, it was mentioned that the whole life has changed during the COVID-19 pandemic and these changes have also influenced the exposure to different chemicals—in some cases, the exposure has increased, but decreased in others. As an example of increased exposure, focus group participants in all countries mentioned the use of disinfectants and cleaning agents. Participants were concerned not only about the amount of cleaning and disinfecting agents used, and their impact on skin, but also about the possibility of breathing them in and their transfer to wastewater.

In addition, when looking at the other sources of environmental pollution, the increased amount of plastic waste due to the changes in business principles of the food industry was mentioned. During the first wave of the COVID-19 pandemic restaurants and other food services offered only takeaway and deliveries packed in single-use plastic packaging. Although such a situation existed in all countries, only one participant in Israel raised this concern.

*“On one hand we wear masks and that may reduce exposure to air pollution. Maybe by working at home we are exposed to less chemicals? Since we are using more disinfectants and eating from single-use plastic more, we are more exposed to chemicals. It is too early to know how COVID impacted our exposure to chemicals….”*

(Israel)

Among examples with lower exposure, inhalation of cleaner air was stressed. In two countries (Denmark and Latvia) less traffic and aviation were reported.

*“Yes, it was just about the corona pandemic and stuff like that. I live in the city where they report that the air pollution inside the city was much less than before because there were simply fewer cars on the roads”*

(Denmark)

In addition, one participant also noted the positive effect of the use of personal protective equipment for the protection of airways (respirators)—originally during the COVID-19 pandemic they are used to mitigate the spreading of the SARS-CoV-2 virus, but they are also regarded to reduce the amount of inhaled dust and chemicals. 

## 4. Discussion

Previous research on dissemination of the results on human biomonitoring in the European Union and worldwide has concluded that the awareness of human biomonitoring and its potential has been raised [17]. However, our results show that the awareness of this topic is diverse and insufficient in countries participating in the second round of the HBM4EU focus group discussions. These results are consistent with the observations from the focus group discussions of the first round of the HBM4EU, where several participants demonstrated some level of understanding of human biomonitoring [7]. Therefore, the results from each of the focus group discussions of the second round of the HBM4EU provide valuable contributions to support the design, implementation, and communication of future biomonitoring actions, as well as to bridge the gap between science and citizens.

Overall, the results of the focus group discussions highlight the high concern of the Europeans about the contaminated and unhealthy food chain and environment. Focus group participants were aware of, and articulated general concern about, the potential uptake of chemicals through food consumption (e.g., preservatives, flavor enhancers, coloring agents, pesticides, herbicides, insecticides, fertilizers, metals) and/or drinking water as well as the exposure from polluted air and water. The participants of the second round of the focus groups of HBM4EU were less aware and concerned about exposure through dermal absorption, when considering all uptake routes (inhalation, ingestion, dermal absorption) that are relevant, in general, for human biomonitoring.

When looking at the expectations of human biomonitoring and the HBM4EU project, continuous research that is better coordinated at both the national and the European levels is encouraged. Although there are some human biomonitoring initiatives in Europe, a harmonized, coordinated, and sustainable European approach is currently still lacking [2]. HBM4EU will be able to provide a baseline against which to measure exposure.

Focus group participants pointed out the need for strict international and national regulations regarding the protection of health and the environment. Policymakers and scientists must work together in the science-policy domain. On the one hand, scientists’ findings should be integrated into relevant legislation when formulating environmental and health policies. On the other hand, scientists should be aware of legislation’s timelines for appropriate submission of scientific results for uptake to policy. 

It seems that the promotion of human biomonitoring and successful implementation of the obtained results in a timely manner can be achieved only when sufficient care is taken regarding the protection of personal data and by a team consisting of multiple professionals with various backgrounds consisting of the national research community from different scientific fields (chemists, biochemists, biologists, experts in biotechnology, toxicologists, physicians, social scientists, nature scientists, psychologists, sociologists, etc.). The focus group participants stressed the need for greater involvement of social scientists which is consistent with previous research [17]. But the role of the governments in the communication of the results was a topic revealing opposite opinions. Most probably such findings are related to the level of trust that citizens feel in their governments. Similar to the results of the discussions of the first round of the HBM4EU focus groups, trust was addressed as a major concern in the second round [7]. However, additional aspects of trust arose during the discussions of the second round. While in the first round some participants mentioned governmental protection of industries, distrust in politicians, and political lobbying, during the second-round participants stressed that private organizations with commercial interests should not be involved in human biomonitoring due to data privacy issues. Data protection issues are also a concern in relation to the use of human biomonitoring in the work environment and the use of the results by the employer.

One of the very positive aspects identified throughout the focus groups’ discussions is related to a high interest in more information and targeted education on changing behavior and personal choice. The behavioral changes require the active involvement of the society (e.g., commuting habits, energy choices, waste disposal, dietary habits, etc.), but they can be empowered only by better knowledge and understanding of chemical exposure, particularly of harmful substances, their sources, routes of exposure and possible adverse health effects. This requires that individuals can grasp the complexity and usefulness of information, including new technologies, legal interventions, or behavioral changes [18,19]. In addition, the content should be changed and adapted to changing life. For, example, the COVID-19 pandemic should be taken into account. Safer and more sustainable handling practices and disinfecting techniques, enhanced monitoring methods, effective communication, and choice of eco-friendly and safer alternatives can be used as examples during the pandemic [11]. 

Traditionally, communication between researchers and society has been one-way, where the public’s perception and understanding are not taken into account [17]. Therefore, the results of these focus group discussions are important to improve both; the content of the communication and the channels used for the communication. It is essential to mention, that in most cases focus group participants were not able to set a borderline between communication on chemical exposure and human biomonitoring. Therefore, it seems that information on human biomonitoring should be shared, as part of the information on chemical exposure and its effect on human health and the environment. In addition, it should be pointed out that the advised approach was very diverse. This would mean that tailor-made messages taking into account country-specific context should be used for the communication on human biomonitoring and HBM4EU. However, transparent, clear, and trustful information and use of non-scientific language was advised throughout all focus groups.

One of the actions to be taken is mainstreaming of related topics into school curricula. Such an approach has been stressed as an effective tool in focus group discussions and it has been used in a related area—occupational health and safety. The European Agency for Safety and Health at Work has prepared several lesson plans based on animations with the character Napo [20]. According to our results, the lesson plans for schoolchildren should be prepared on the topics which are of main concern—exposure to chemicals from food and aspects related to personal behavior and choice which may influence the level of exposure. 

Raising awareness amongst the general public, including children, may promote the engagement of citizens, which in turn may empower them to put pressure on politicians to take effective actions in the area of chemical safety. One of the effective ways is the promotion of work of non-governmental organizations. The results of our research show that the focus group participants which were active members of non-governmental organizations (e.g., in North Macedonia) were better aware of problems related to the environment and also could give examples of the success of non-governmental organizations (e.g., in Israel). The experience in EU countries shows that citizens can effectively influence politicians via non-governmental organizations to take action to reduce the exposure of the general public to chemicals. For example, in 2017 European non-governmental organizations called on dentists to ban the use of mercury in children [21]. 

When discussing the effects of the COVID-19 pandemic, focus group participants mainly focused on increased use of disinfectants and cleaning agents and reduced air pollution from lower activity in the industry, aviation, and road traffic. Only one participant mentioned an increased amount of waste due to the packaging of take-away food and medical waste. Such results can be explained by the fact that disinfectants and cleaning agents were used by every single member of the society and washing, and disinfection of hands and surfaces was used as one of the messages at the early stages of the pandemic to mitigate the spread of the virus. 

Several limitations of our study were identified. One of them is related to the characteristics of the recruited focus group participants. Although in general, participants who were engaged in focus groups of the second round of HBM4EU do vary in the sociodemographic background, participants with a university degree seem to be overrepresented. Some of the research teams (e.g., Israel and Latvia) faced problems while trying to recruit focus group participants to have a representative sample of relevant nations. Thus, in Israel, the participants primarily were well–educated females, but in Latvia—educated males. Similarly, to the focus groups carried out during the first round of the HBM4EU focus groups, individuals with lower education were underrepresented in the focus groups [7]. As previous studies have reported that perceptions of environmental issues, such as air quality, are affected by education level, age, and gender, further research is needed to understand perceptions and concerns among lower socioeconomic groups [18,22,23]. This might require the adaption of research methods (e.g., use of different criteria for recruitment of the participants, modification/simplification of guidelines, etc.). In addition, a sufficient proficiency level of the national language was an entrance criterion to participate in the focus group discussions, therefore the opinion of the communities without sufficient language proficiency is not represented in this survey. It might have affected the results of the focus group discussions as it has already been described that minority residents might exhibit lower levels of risk perception compared to the general population [24].

Another limitation is related to the flexibility of the content of the second round of the HBM4EU focus group discussions. Although the focus groups followed standardized guidelines, the national researchers were allowed to adapt the content to the national context. There were also situations when already during the discussions the moderator tried to avoid deeper discussions of some of the “sensitive” topics (e.g., in Latvia, due to the fact that some of the participants focused on conspiracy theories). This has resulted in the fact that some of the topics were not sufficiently covered in all participating countries (see Table A1 in Appendix A). The section on the perception of changes during the COVID-19 pandemic has been especially affected, as this topic has been covered only by three countries—Hungary, Israel, and Latvia. Although the results obtained from these countries provide valuable insight into the opinion and concerns in the society, they cannot be attributed to the general perception of all Europeans due to the fact that there are groups of countries not represented (e.g., Northern countries, so-called “old” member states of the European Union). Therefore, this topic should be addressed in the future as it could provide additional knowledge to improve preparedness for the management of future pandemics.

There was also variation in the length of each focus group (70–180 min) and the number of participants per session (3–12). Although experienced focus group moderators tried to address each participant individually and provide equal opportunities, the online environment which was used in some countries as the result of differing national COVID-19 restrictions turned out to be a challenge. Non-uniformity in focus groups might have impacted online discussions rather than on-site. In addition, some countries (e.g., North Macedonia) reported that most of the respondents were more active and more involved in the first part of the discussion which was dedicated to chemical exposures, while in the second part when human biomonitoring was discussed, those with some prior knowledge of the topic were more active.

Despite these limitations, we believe that our results provide valuable information on the understanding of citizens’ perceptions, awareness, and concerns about the exposure of chemicals in their daily life and environment and the future of human biomonitoring. There is a need and support from the European citizens for further research in the area of human biomonitoring and perception of chemical exposure. At the same time, further analyses of the existing data should be done to identify if there exists any influence of geographical location or historical background of the country on the perception and concerns of the citizens. In addition, further research should be done on the impact of the COVID-19 pandemic on the exposure of citizens to chemicals. This might serve as a basis for further policy implications in the protection of the European citizens and better preparedness for the management of possible pandemics in the future.

## 5. Conclusions

The main concerns about the exposure to chemicals revealed by the focus group participants are related to the potential uptake of chemicals through food consumption and/or drinking water as well as the exposure to chemicals in the environment from polluted air and water. The COVID-19 pandemic has also influenced the perception of such exposure– in some cases, the exposure has increased (e.g., use of disinfectants and cleaning agents), but in others—decreased (e.g., air pollution from industry and traffic). 

This study provides data on the clear need for further research and policy uptake in the field of human biomonitoring, which should be coordinated and harmonized at the European level. Based on the research result, other activities should be carried out. The main directions of such activities should focus on raising awareness amongst the general public, implementation of policy measures, and mainstreaming of topics related to chemical exposure and human biomonitoring into the education system. As most focus group participants were not able to set a borderline between chemical exposure and human biomonitoring, an integrated multi-sectoral approach harmonized at the European level should be used, taking into account country-specific context in the field of chemical exposure and human biomonitoring. Raising awareness amongst the general public may promote the engagement of citizens, which in turn may empower them to put pressure on politicians to take effective actions. 

## Figures and Tables

**Table 1:** Description of the focus groups discussions by country.

Country	Number of Sessions	Total Sample Size	Length of Session (min)	Compensation	Form of Discussions
Cyprus	1	10	180	Yes	Onsite
Denmark	2	13	180	Yes	1 onsite 1 online (Brainstork)
The Netherlands	4	16	70–90	Yes	Online (GoToMeeting)
Hungary	1	11	80	No	Online (GoToMeeting)
North Macedonia	1	12	120	No	Online (Zoom)
Israel	1	8	90	Yes	Online (Zoom)
Latvia	1	8	115	No	Online (Zoom)
Total	11	78			

**Table 2:** Sample characteristics by country.

Country	Gender	Age Range	Remarks Regarding Background
Cyprus	5 females 5 males	25–45	Various professions (not recorded in detail)
Denmark (2 groups)	7 females 6 males	19–70	Teachers, social workers, workers in financial and IT sector, printer, carpenter, employer, students, retired persons, unemployed.
The Netherlands (4 groups)	8 females 8 males	24–60	Homemakers, security worker, students, data analyst, (medical) administrative workers, the Navy, IT worker, campaign director, artist, technical specialist.
Hungary	6 females 5 males	18–74	Teacher, musician, engineers, employees with a natural science degree, office assistants.
North Macedonia	6 females 6 males	21–72	Chemist, medical practitioner, biologist, people working in the field of living environment protection and/or accreditation of laboratories, two young eco-activists in the field of air quality and waste management, young pregnant women, sports and health teacher, IT student, two highly motivated citizens.
Israel	7 females 1 male	23–58	Lawyer, doctoral student (environmental engineering), gardener, director of environment and sustainability at a non-governmental organization, environmental volunteer and activist, teacher, epidemiologist, an employee at a start-up company.
Latvia	2 females 6 males	31–66	Occupational health and safety expert, IT expert, client consultant, an employee in a museum, teacher, retired person (former interpreter), unemployed person, policeman.

**Table 3:** Categories and subcategories identified during the research analysis.

Subcategory	Cyprus (*n* = 10)	Denmark (*n* = 13)	The Netherlands (*n* = 16)	Hungary (*n* = 11)	North Macedonia (*n* = 12)	Israel (*n* = 8)	Latvia (*n* = 8)	In Total (*n* = 78)
**1. Perception of chemical risks and their impact on human health**								
**1.1. Main concerns regarding exposure and health effects**								
**General sources of exposure**								
Chemicals in food	*++*	*++*	*++*	*n.c.*	*++*	*+*	*-*	9
Air pollution	*+*	*+*	*+*	*n.c.*	*++*	*+*	*-*	6
Chemicals in drinking water	*-*	*++*	*-*	*n.c.*	*++*	*-*	*-*	4
General environmental pollution	+	*+*	*-*	*n.c.*	*-*	*+*	*-*	3
Chemicals and dust in the workplace	*-*	*+*	*-*	*n.c.*	*+*	*+*	*-*	3
Personal care products	*-*	*-*	*++*	*n.c.*	*-*	*-*	*-*	2
Exposure from several sources	*-*	*+*	*-*	*n.c.*	*-*	*-*	*+*	2
**Specific sources of exposure**								
Air pollution from traffic	*+*	*+*	*+*	*n.c.*	*+*	*-*	*-*	4
Living in/close to a polluted area	*++*	*-*	*-*	*n.c.*	*+*	*+*	*-*	4
Air pollution from industry	*-*	*-*	*+*	*n.c.*	*+*	*-*	*-*	2
Plantations contaminated by pesticides or herbicides	*+*	*-*	*-*	*n.c.*	*+*	*-*	*-*	2
Chemicals from food products	*+*	*+*	*-*	*n.c.*	*-*	*-*	*-*	2
Storage of hazardous chemicals	*-*	*-*	*-*	*n.c.*	*+*	*+*	*-*	2
**Exposure routes of chemicals**								
Oral uptakes of harmful substances	*++*	*-*	*-*	*-*	*+*	*-*	*-*	3
Inhalation of contaminated air	*+*	*-*	*-*	*-*	*+*	*-*	*-*	2
Exposure through skin contact	*+*	*-*	*-*	*-*	*+*	*-*	*-*	2
Substances from several sources	*-*	*++*	*-*	*-*	*-*	*-*	*-*	2
**Health effects**								
Cancer	*+*	*+*	*-*	*n.c.*	*+*	*-*	*+*	4
Allergy	*-*	*+*	*-*	*n.c.*	*-*	*-*	*++*	3
Long term effects (in general)	*-*	*+*	*+*	*n.c.*	*+*	*-*	*-*	3
Skin problems	*-*	*+*	*-*	*n.c.*	*+*	*-*	*+*	3
Respiratory diseases	*-*	*-*	*-*	*n.c.*	*+*	*-*	*+*	2
Cardiovascular diseases	*-*	*-*	*-*	*n.c.*	*+*	*-*	*+*	2
Fertility	*-*	*+*	*-*	*n.c.*	*+*	*-*	*-*	2
**Factors promoting interest**								
‘Well-known’ diseases (e.g., cancer)	*+*	*+*	*-*	*-*	*n.c.*	*n.c.*	*n.c.*	2
Previous experience	*+*	*-*	*-*	*+*	*n.c.*	*n.c.*	*n.c.*	2
Availability of healthier alternatives/choices	*-*	*-*	*+*	*+*	*n.c.*	*n.c.*	*n.c.*	2
**1.2. Concerns regarding harmful substances with the most exposure**								
Food preservatives, flavor enhancers, coloring agents / E-numbers	*-*	*++*	*++*	*-*	*+*	*-*	*++*	7
Pesticides, herbicides, insecticides	*+*	*+*	*+*	*-*	*+*	*-*	*+*	5
Heavy metals	*+*	*-*	*-*	*-*	*+*	*+*	*+*	4
Dioxins, polychlorinated biphenyls	*-*	*-*	*-*	*-*	*+*	*-*	*+*	2
Cosmetics	*+*	*+*	*-*	*-*	*-*	*-*	*-*	2
Fertilizers	*+*	*-*	*-*	*-*	*-*	*-*	*+*	2
Pharmaceutical drugs, medication	*+*	*+*	*-*	*-*	*-*	*-*	*-*	2
Smoking and tobacco products	*+*	*-*	*-*	*-*	*-*	*-*	*+*	2
Substances released during the combustion process	*-*	*-*	*-*	*-*	*-*	*+*	*+*	2
Petroleum products	*-*	*-*	*-*	*-*	*+*	*-*	*+*	2
**1.3. Aspects related to personal behavior**								
Personal efforts to avoid chemicals								
Consuming biological and organic food	*+*	*++*	*+*	*n.c.*	*n.c.*	*n.c.*	*n.c.*	4
Getting fresh food	*-*	*+*	*+*	*n.c.*	*n.c.*	*n.c.*	*n.c.*	2
Active avoidance of E-numbers	*-*	*-*	*++*	*n.c.*	*n.c.*	*n.c.*	*n.c.*	2
Factors influencing personal behavior								
Economic factors	*-*	*+*	*-*	*+*	*n.c.*	*n.c.*	*n.c.*	2
Low awareness	*-*	*-*	*-*	*+*	*n.c.*	*n.c.*	*n.c.*	1
**2. Being familiar with human biomonitoring and HBM4EU**								
**2.1. Perception of human biomonitoring**								
Have not heard about human biomonitoring/ unknown term/very low and limited understanding	*++*	*++*	*++*	*++*	*+*	*n.c.*	*++*	11
Analysis of biological samples from the human body (in general)	*+*	*+*	*+*	*+*	*+*	*n.c.*	*-*	5
Analysis of blood	*+*	*+*	*-*	*-*	*+*	*n.c.*	*-*	3
Analysis of urine	*+*	*-*	*-*	*-*	*+*	*n.c.*	*-*	2
Analysis of semen	*-*	*+*	*-*	*-*	*-*	*n.c.*	*-*	1
Analysis of hair	*-*	*+*	*-*	*-*	*-*	*n.c.*	*-*	1
Monitoring of the presence/limits of chemicals	*+*	*+*	*-*	*-*	*+*	*n.c.*	*-*	3
Cocktail effect	*-*	*+*	*+*	*-*	*-*	*n.c.*	*-*	2
**2.2. Actors in human biomonitoring**								
Multiple professionals	*+*	*+*	*+*	*+*	*+*	*n.c.*	*n.c.*	5
State authorities (in the field of health)	*+*	*+*	*+*	*-*	*-*	*n.c.*	*n.c.*	3
National scientific institutions, researchers from different scientific fields	*++*	*+*	*-*	*-*	*-*	*n.c.*	*n.c.*	3
Politicians, Parliament	*+*	*+*	*-*	*-*	*-*	*n.c.*	*n.c.*	2
Sports industry	*-*	*+*	*-*	*-*	*+*	*n.c.*	*n.c.*	2
Laboratories	*-*	*-*	*-*	*+*	*+*	*n.c.*	*n.c.*	2
**3. Expectations from human biomonitoring and particularly HBM4EU**								
Policy-related actions	*+*	*+*	*-*	*+*	*+*	*+*	*-*	5
Getting more control and legislation in the field	*++*	*+*	*-*	*+*	*-*	*-*	*+*	5
Better research in the field/human biomonitoring done systematically	+	*+*	*-*	*-*	*++*	*-*	*-*	4
Better labelling of products, more information on labelling	*+*	*+*	*-*	*+*	*-*	*-*	*-*	3
Expanding the list/range of hazardous chemicals to be measured/monitored	*-*	*-*	*-*	*-*	*+*	*-*	*+*	2
Vulnerable groups	*+*	*-*	*-*	*-*	*-*	*-*	*+*	2
Additional initiatives	*+*	*+*	*-*	*-*	*-*	*-*	*-*	2
Mainstreaming of the relevant topics in school curricula	*+*	*-*	*+*	*-*	*-*	*-*	*-*	2
**4. Communication on human biomonitoring and HBM4EU**								
**4.1. Content of communication**								
Understandable, clear, a non-scientific manner	*++*	*++*	*+*	*-*	*-*	*+*	*++*	8
Selected/targeted information	*+*	*-*	*-*	*+*	*+*	*-*	*-*	3
Communication from governments	*-*	*+*	*-*	*-*	*+*	*-*	*+*	3
Personalized with the context of choice	*+*	*-*	*+*	*-*	*-*	*+*	*-*	3
Communication with a positive message	*-*	*-*	*-*	*+*	*-*	*+*	*-*	2
Intense and powerful communication	+	*-*	*-*	*+*	*-*	*-*	*-*	2
Detailed procedural information on HBM4EU	*+*	*-*	*+*	*-*	*-*	*-*	*-*	2
**4.2. Communication channels**								
Mass media campaign (regular media, TV)	*+*	*+*	*-*	*+*	*+*	*-*	*-*	4
Social media	*+*	*-*	*-*	*+*	*+*	*-*	*+*	4
Apps, tools, kits	+	*+*	*-*	*-*	*+*	*+*	*-*	4
Special events	+	*-*	*-*	*+*	*-*	*+*	*-*	3
Special website	+	*-*	*-*	*+*	*-*	*-*	*-*	2
Simultaneous use of different channels	*+*	*-*	*-*	*+*	*-*	*-*	*-*	2
Visual and graphical display of results (infographics, videos)	*+*	*-*	*-*	*-*	*+*	*-*	*-*	2
**5. COVID-19 and use of chemicals**								
Increased use of disinfectants and cleaning agents	*n.c.*	*n.c.*	*n.c.*	*+*	*n.c.*	*+*	*+*	3
Fewer emissions from transport and aviation/less air pollution	*n.c.*	*n.c.*	*n.c.*	*+*	*n.c.*	*+*	*+*	3
Time proportion spent inside and outside	*n.c.*	*n.c.*	*n.c.*	*-*	*n.c.*	*+*	*+*	2

“++”—mentioned by most /several participants; “+”—mentioned by one, two or some participants; “-“—nobody has mentioned this topic; n-number of focus group participants; *n.c*.—the topic was not covered during the particular focus group discussion.

## Data Availability

Data sharing of focus group discussions is not applicable as the data consists of focus group transcripts, which for reasons of confidentiality, cannot be shared.

## Appendix A

**Table A1:** The most relevant quotes to support the categories.

Supporting Quote	Country
1. Perception of chemical risks and their impact on human health	
1.1. Main concerns regarding exposure and health effects	
“*To which chemicals are we exposed the most? … I would say, that I have difficulties to answer as they are all around us, we are exposed everywhere*”	Latvia
“*Food is another source of exposure to chemicals and as an example, I would like to mention the consumption of the food color that is found in E101 (sunset yellow) juices …. This is a very carcinogenic chemical*”	North Macedonia
“*As a resident of Haifa Bay [polluted are in Israel], I am very aware of air pollution problems and think human biomonitoring is relevant to my life*”	Israel
*“I simply believe that it is all of it. I think it can be our clothes, the way it is produced, the substances in it, what we wash it in, the food we consume, it can be something in the air and ordinary pollution from factories“*	Denmark
*“On account of serious health issues such as cancer… especially with regards to what we eat and how we eat it”*	Cyprus
*“Health problems…. Yes, I have not been subjected to them, but it seems, I can’t call it knowledge, more suspicions, I have heard, that allergic reaction seems to be more often observed in children“*	Latvia
*“Yes, the matter worries me a lot because it endangers human health and relates to the more frequent incidences of cancer and other severe diseases, which are observed”*	Cyprus
*“I mean, when you go somewhere with your whole family every year, you would want to know, is the air, is the food, the environment there healthy? If you go there on vacation every year, you can also choose for another destination”*	The Netherlands
1.2. Concerns Regarding Harmful Substances with the Most Exposure	
“*I also think that it is the substances we intake, I cannot say which ones, but I think that it is through our food and our drinks and such. There are coloring agents, preservatives, these are some of the most primary*”	Denmark
*“Pesticides can contaminate our food, the water and the environment... I remember reading as a student in Mytilene [Greece] about a study, which showed that the pesticides sprayed on the local olive groves contaminated the soil and water bodies and that the contamination persisted for a long time. I mean, the cycle of pesticides ‘use and effects’ is unbelievable”*	Cyprus
“*I am very concerned about heavy metals in food supplements. I don’t think this is sufficiently regulated*”	Israel
1.3. Aspects Related to Personal Behavior	
*“I personally tried and planted some things [fruit/trees/vegetables] at my house so that I do not eat chemicals from supermarkets etc.*”	Cyprus
“*When I see that I can get a food that costs roughly the same and which is organic, then I buy the organic if there is not that big a price difference*”	Denmark
2. Present Situation of Human Biomonitoring	
2.1. Being Familiar with Human Biomonitoring and HBM4EU	
“*That what comes into my mind on human biomonitoring at once is … more related with medical parameters, for example, the oxygen concentration in blood, or blood pressure, or something like that*”	Latvia
*“… I have heard the term … before, primarily due to work. I did for my diploma thesis at university. It means using human blood or urine to measure chemicals in a lab”*	Cyprus
*“… for example, examinations of heavy metals in samples of human origin”*	North Macedonia
“*Yes, so you will find it e.g., about the inspection of semen quality where it is said that in general, it (semen quality) has got worse and worse over the years due to lifestyle, among other things, right*”	Denmark
2.2. Actors in Human Biomonitoring	
”*It shouldn’t be only one type of competent experts, who are responsible, but a combination of different experts, such as sociologists, biochemists, and health professionals…”*	Cyprus
*“.., you really should prevent misuse [of HBM data], for example by insurance companies or others who could possibly (mis)use these data for their own purposes”*	The Netherlands
“...*but it also depends on the type of company that you are working for, and what the societal importance is for the company to demand participation in HBM studies”*	The Netherlands
3. Expectations from Human Biomonitoring and Particularly HBM4EU	
*“I was also thinking about how politicians could be influenced. I also think that without real political support it is very hard to achieve meaningful change…”*	Hungary
“*Yes, I like such studies…. [they should] serve as a base to improve legislation and the guidelines or some other things in many areas… in the area of medicine and food… An important aspect is the education of the society, I think, that would be one, if not the result, then at least the aim of this project*“	Latvia
*“But just some legislation that makes some products just not legal at all and you are not allowed to sell them and just do it for the whole EU so you can’t just drive down to the border in Germany and then take it home as a product.”*	Denmark
“*This is a good step for cooperation between science and politics. This project ‘diagnoses’ the condition, which is the most important, and it leads to successful ‘treatment’”*	North Macedonia
*“Systematic monitoring should be done. The Council of Ministers or a specific ministry should have the responsibility to undertake it. …There should be cycles of systematic awareness-raising every 1-2 years, where overall results are communicated to citizens so that they can have a visual understanding of the situation”*	Cyprus
*“In order for laboratories to work, it is necessary to have interest from those who need these measurements, for example potential polluters*”	North Macedonia
“*The case is that if a product is approved in EU- countries, then it can be sold in all countries. So, of course, it is important to raise the standards for the entire EU*”	Denmark
*“Human biomonitoring should be introduced to high-school students as part of a lesson or seminar. For example, we were introduced to the importance of blood donations as secondary-school students. Since then, we became blood donors, again voluntarily. So, the message is delivered to the student at that age, then it will be easier for him/her to participate in human biomonitoring studies in the future”*	Cyprus
*“You could make a requirement that all supermarkets should have a section with everyday products, that do not contain chemicals and are 100% approved in the field of something. I mean that kind of initiative would be relatively easy to implement without changing so much”*	Denmark
4. Communication of the Results of Human Biomonitoring and HBM4EU	
4.1. Content of Communication	
“*…it is very important to raise awareness among the public and maybe then they could put pressure on policymakers. It is very difficult to achieve anything without political support*”	Hungary
“*I also think it would be best if it was published in e.g., an article in the media or something with source references to this report. So, kind of … understandable for the common man who is not a researcher*”	Denmark
*“… I think it is the most appropriate to target parents of a certain age group of children and young people, through social media posts and videos, which will talk about children’s health*”	North Macedonia
“*It is important to target young children and increase awareness about environmental health. As an environmental activist, we targeted schools and kindergartens in messaging about single-use plastic and have been successful*”	Israel
“*Look, there is of course not much you can do about air pollution. But if you know, for example, that if you use that washing product or that cosmetic, you will already get less or no chemicals. I think if there is more clarity and information about that, then you can already do things for yourself that you can influence. So that people become more aware of their responsibility”*	The Netherlands
“…. *I’d disseminate positive information as clearly and strikingly as possible. In the long-term, a label should be used that would get a (coherent) E-label and inform us if we bought the healthiest or the least harmful product… Definitely, a positive campaign is needed*”	Hungary
“*I would want to know upfront, what they are going to do [with the samples] and what they are going to research*”	The Netherlands
4.2. Communication Channels and Methods	
“*I also think it is important to reach as many age groups and people as possible and social media platforms are the best tools for this. I’d also rather use surprising or more effective advertisements or campaigns that would shock people a little*”	Hungary
“*A component of the follow-up project should provide communication, information, and dissemination of results, in the form of a media campaign, with involvement of the NGO sector*”	North Macedonia
*“… information should not come from the top, from the government, … it should more come from “side”…*”	Latvia
5. Perception of Influence of the COVID-19 Pandemic on Exposure to Chemicals	
“*I’d like to mention that all these disinfectants that we now pump into the water and everything, how much can these be chemically cleaned? I don’t think it’s a brilliant idea to disinfect absolutely everything, I think that’s dumb. We could use fewer disinfectants to achieve our goal; however, people use tons of disinfectants. I don’t think that’s very smart*”	Hungary
“*On one hand we wear masks and that may reduce exposure to air pollution. Maybe by working at home we are exposed to less chemicals? Since we are using more disinfectants and eating from single-use plastic more, we are more exposed to chemicals. It is too early to know how COVID impacted our exposure to chemicals….”*	Israel
*“Yes, it was just about the corona pandemic and stuff like that. I live in the city where they report that the air pollution inside the city was much less than before because there were simply fewer cars on the roads”*	Denmark
“*Firstly, it seems to me when this madness started, immediately the number of flights went down, let’s say, the air became cleaner*“	Latvia
